# Oxysterols in the Immune Response to Bacterial and Viral Infections

**DOI:** 10.3390/cells11020201

**Published:** 2022-01-07

**Authors:** Cheng Xiang Foo, Stacey Bartlett, Katharina Ronacher

**Affiliations:** 1 Translational Research Institute, Mater Research Institute, The University of Queensland, Brisbane, QLD 4102, Australia; chengxiang.foo@uq.net.au (C.X.F.); stacey.bartlett@mater.uq.edu.au (S.B.); 2 Australian Infectious Diseases Research Centre, The University of Queensland, Brisbane, QLD 4072, Australia

**Keywords:** oxysterols, oxidized cholesterol, bacteria, virus, infection, *M. tuberculosis*, SARS-CoV-2, GPR183, 25-hydroxycholesterol, 7α,25-dihydroxycholesterol, cholesterol 25 hydroxylase

## Abstract

Oxidized cholesterols, the so-called oxysterols, are widely known to regulate cholesterol homeostasis. However, more recently oxysterols have emerged as important lipid mediators in the response to both bacterial and viral infections. This review summarizes our current knowledge of selected oxysterols and their receptors in the control of intracellular bacterial growth as well as viral entry into the host cell and viral replication. Lastly, we briefly discuss the potential of oxysterols and their receptors as drug targets for infectious and inflammatory diseases.

## 1. Introduction

Cholesterol is a ubiquitous sterol that is synthesized by mammalian cells and forms an essential component of the cell membrane [1]. It is involved in a variety of cellular processes, ranging from controlling membrane permeability and integrity to cellular signaling. In addition, cholesterol also serves as an important precursor for several molecules, such as steroid hormones, bile acid, vitamin D, and oxysterols [2]. Oxysterols are formed by the hydroxylation of cholesterol by enzymes or pro-oxidants and are important for regulating cholesterol homeostasis. In recent years, oxysterols have also emerged as important bioactive molecules in immunity and inflammation, with multiple immunoregulatory functions across several different cell types and organs. However, the actions and implications of oxysterols in the immune response to bacterial and viral pathogens are largely underexplored. Here, we review the literature and summarize the currently available knowledge on the role of oxysterols in the immune response to infectious pathogens of both bacterial and viral origin. Clear evidence exists that some oxysterols possess anti-microbial and anti-viral activities, mostly through the modulation of the host immune response. Lastly, we propose and provide evidence that this group of lipid mediators and their respective receptors may be targeted pharmacologically to improve the treatment outcomes of infectious diseases.

## 2. Types of Oxysterols and Their Receptors

**Figure 1:**
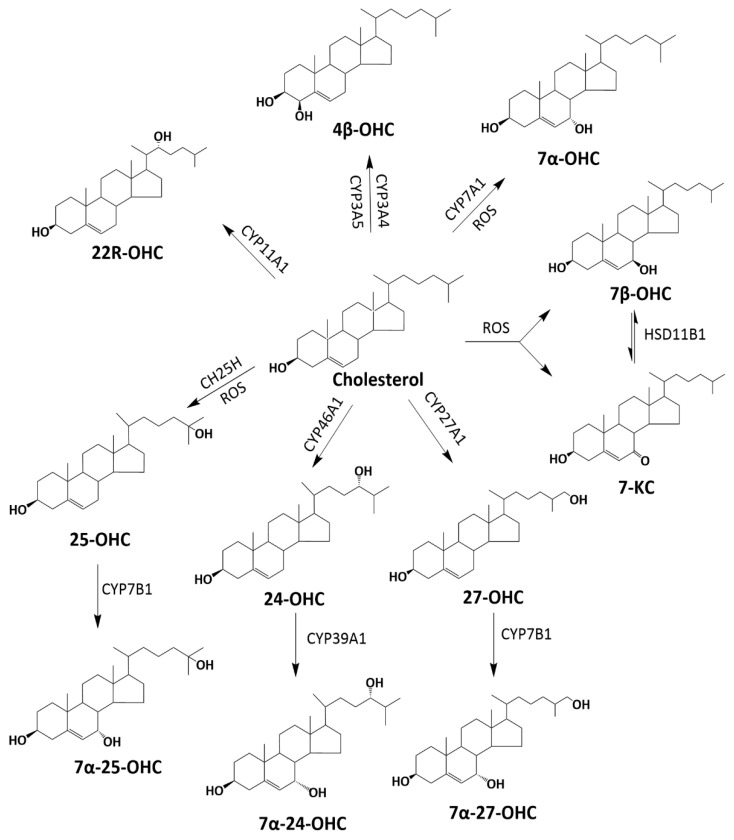
Overview of the generation of selected oxysterols from cholesterol. Oxysterols are generated from cholesterol through oxidation by pro-oxidants or enzymes. The majority of these enzymes involved in the synthesis of oxysterols are from the family of cytochrome P450 enzymes [3,5], with the exception of cholesterol 25 hydroxylase (CH25H).

Structurally similar to cholesterol but with additional oxygen-containing functional groups, oxysterols are oxidized cholesterols that can either be derived from the diet or produced endogenously [3]. Dietary sources of oxysterols include cholesterol-rich foods such as meats, eggs, and dairy products [4]. Endogenously, oxysterols are derived through the oxidation of cholesterol by enzymes or reactive oxygen species (ROS) (Figure 1) [5]. The majority of enzymes involved in the synthesis of oxysterols belong to the family of cytochrome P450 enzymes [3,5], with the exception of cholesterol 25 hydroxylase (CH25H), which belongs to the family of enzymes that utilize oxygen and diiron cofactors for hydroxylation [6].

Oxysterols are present at low concentrations, up to 5-fold lower compared to cholesterol, in circulation [7], and were initially thought to only play a role in regulating cholesterol homeostasis. Oxidization makes cholesterol more hydrophilic, thereby facilitating its elimination. In addition, oxysterols act as a negative feedback control in response to high cholesterol concentrations [8] and can restore homeostasis [9]. Oxysterols are known to activate the transcriptional regulators of cholesterol synthesis—namely, the sterol regulatory element binding protein (SREBP) and the Liver X receptors (LXRs) [9,10]. In response to low cholesterol concentrations, SREBP is translated in the ER membranes and transported to the Golgi complex with the help of the SREBP cleavage activating protein (SCAP) for cleavage. The cleaved SREBP is subsequently able to enter the nucleus, triggering the transcription of genes involved in cholesterol synthesis [11,12]. In contrast, when cholesterol levels are high, cholesterol binds to SCAP, which in turns binds to insulin-induced gene 1 protein (INSIG1) and INSIG2, which are ER retention proteins, thereby preventing SREBP activation [12]. Oxysterols have shown to directly bind to and activate INSIGs, thereby preventing the activation and cleavage of SREBP, leading to the downregulation of cholesterol synthesis [8,11,12]. In addition, oxysterols are also ligands for LXRs that exist in two isoforms, LXRα and LXRβ [8]. In response to high cholesterol concentrations, the activation of LXR by oxysterols leads to the upregulation of the genes involved in lipid metabolism, notably genes from the ATP-binding cassette family of membrane transporters, which regulate cholesterol efflux and excretion, resulting in a reduction in intracellular cholesterol accumulation [8,10]. Additionally, oxysterols have been shown to acutely regulate cholesterol concentrations through several post-transcriptional regulatory mechanisms [9]. For instance, several oxysterols, including 25-hydroxycholesterol (25-OHC), alter the enzymatic activity of 3-hydroxy-3-methylglutaryl-CoA reductase (HMGR), an important rate-limiting enzyme involved in the synthesis of mevalonate in the cholesterol synthesis pathway [13]. Some oxysterols can also induce cholesterol esterification through the activation of acyl-CoA cholesterol acyl transferase (ACAT) within the cell, resulting in a rapid reduction in cholesterol [14].

The essential role of oxysterols in governing cholesterol homeostasis has long been established [8]. More recently, several studies have shown the numerous immunological functions of oxysterols, ranging from their involvement in chemotaxis and the development of immune cell niches [15,16,17,18] to skewing immune cell phenotypes and coordinating inflammatory responses [19] (Table 1). In addition, studies have also demonstrated that oxysterols bind to a wide range of receptors, from nuclear receptors such as retinoic acid receptor-related orphan receptors (RORs) to LXRs, estrogen receptors (ERs), and transmembrane G-protein coupled receptors (GPCRs) to carry out their diverse immunological functions [20]. This review focuses on the role oxysterols play in the host response to bacterial and viral infections.

## 3. Oxysterols in Bacterial Infections

Over the past decade, various studies have elucidated the link between oxysterols and the innate immune response to intracellular bacteria. An increased susceptibility to infection by *Listeria monocytogenes* and *Mycobacterium tuberculosis* was observed in LXR knock-out (KO) models, which provided potential evidence of the involvement of oxysterols in the immune response to these infections, given that oxysterols are ligands for LXR receptors [59,60]. Indeed, two recent studies have demonstrated an immunomodulatory role of the oxysterol 25-OHC against intracellular bacteria and secreted bacterial toxins [31,32]. The first study revealed a role for 25-OHC in the immunity against several intracellular bacteria. The authors demonstrated that in *Listeria monocytogenes* infection, CH25H KO mice had increased bacterial dissemination compared to wild-type (WT) -infected mice [31]. Additionally, the in vivo administration of 25-OHC reduced the bacterial burden, supporting the immunomodulatory role of 25-OHC. Furthermore, using both *L. monocytogenes* and *Shigella flexneri*, the authors demonstrated that the in vitro administration of 25-OHC resulted in the downstream activation of ACAT, which in turn triggers cholesterol remodeling on the plasma membrane and thereby restricts the cell-to-cell dissemination of these pathogens [31]. The second study elucidated a protective role of 25-OHC against cholesterol-dependent cytolysins (CDCs), a pore-forming toxin that is secreted by a variety of pathogenic bacteria [32]. The authors demonstrated in bone marrow-derived macrophages (BMDMs) that interferon (IFN) signaling mediates cholesterol remodeling on plasma membranes through the CH25H/25-OHC axis [32]. The reduced availability of cholesterol on the plasma membrane induced by 25-OHC resulted in a reduced binding of CDCs, thereby conferring resistance to CDC-induced pore damage. These findings were further explored in in vivo models, whereby the authors demonstrated that CH25H deficiency resulted in ulcerative lesions and larger lesion sizes. Additionally, pre-treatment with 25-OHC was found to be protective against CDC-mediated tissue damage [32]. Collectively, both studies showed that 25-OHC modifies the cholesterol content on the plasma membrane, thereby conferring resistance to CDCs as well as bacterial pathogens.

The role of oxysterols in the innate immune response to *M. tuberculosis* has also been investigated [35,61]. IL-36, a newer family of the IL-1 family of cytokines, is produced in macrophages upon *M. tuberculosis* infection to regulate the synthesis of oxysterols such as 25-OHC and 27-hydroxycholesterol (27-OHC) [61]. The production of these oxysterols subsequently led to downstream LXR activation, suppressing cholesterol metabolism and reducing mycobacterial growth. More recently, our laboratory discovered an important role for 7α,25-dihydroxycholesterol (7α,25-OHC) and its receptor GPR183 in *M. tuberculosis* infection [35]. GPR183, also known as Epstein–Barr virus-induced G protein coupled-receptor 2, was discovered in the 1990s as one of the genes that were upregulated upon infection with Epstein–Barr virus in Burkitt’s Lymphoma cell lines [62]. GPR183 is expressed across several types of innate and adaptive immune cells such as dendritic cells, innate lymphoid cells 3 (ILC3s), macrophages, and T and B lymphocytes [5]. Oxysterols are known ligands for GPR183, with 7α,25-OHC being the most potent endogenous agonist [24]. The intracellular growth of both *M. tuberculosis* and *M. bovis* BCG in primary human monocytes was significantly restricted in the presence of 7α,25-OHC, an effect that was abrogated by a specific GPR183 antagonist [35]. This growth inhibitory effect, which was associated with induction of autophagy and the negative regulation of type I IFNs, was specific to primary human monocytes [35] and was not observed by others in a murine macrophage cell line [36]. In patients with pulmonary tuberculosis, lower GPR183 expression in the blood correlated significantly with more severe disease on chest X-ray, and GPR183 KO mice exhibited a higher lung mycobacterial burden and dysregulated type I IFNs in early infection [35], highlighting the important roles of oxysterols and GPR183 in the immune response to mycobacterial infections.

In addition to the immunomodulatory effects, a recent study provided evidence that the enzymes produced by *M. tuberculosis* regulate the oxysterol metabolism to limit the effective induction of the immune response [63]. The mycobacterial enzyme 3β-hydroxysteroid dehydrogenase (3β-HSD) can metabolize 25-OHC and 7α,25-OHC, among others, to render them inactive. As these oxysterols have protective roles in immunity against *M. tuberculosis*, it is possible that 3β-HSD, along with the other identified Mtb enzymes CYP124, CYP125, and CYP142, targets these oxysterols to interfere with and evade the host immune response, thereby allowing its continual persistence in host macrophages [63]. It is also possible that *M. tuberculosis* produces itself antagonists against GPR183, as has been demonstrated for other bacteria. *Eubacterium rectale*, for instance, produces lauroyl tryptamine, which is able to bind to and antagonize GPR183 against its endogenous agonist 7α,25-OHC at a 0.98 μM half-maximal inhibitory concentration [64]. Whether *M. tuberculosis* is able to produce antagonists for GPR183 or other oxysterol receptors remains to be elucidated.

Oxysterol gradients can also facilitate the migration and recruitment of cells. For instance, 7α,25-OHC attracts GPR183-expressing immune cells to secondary lymphoid organs [15,16,17,18]. In the host immune response to *Citrobacter rodentium* infection, 7α,25-OHC production attracts GPR183-expressing ILC3s and GPR183 KO mice have a reduced abundance of IL-22-producing intestinal ILC3s, which is associated with greater disease severity and mortality rates as compared to WT mice [37].

**Figure 2:**
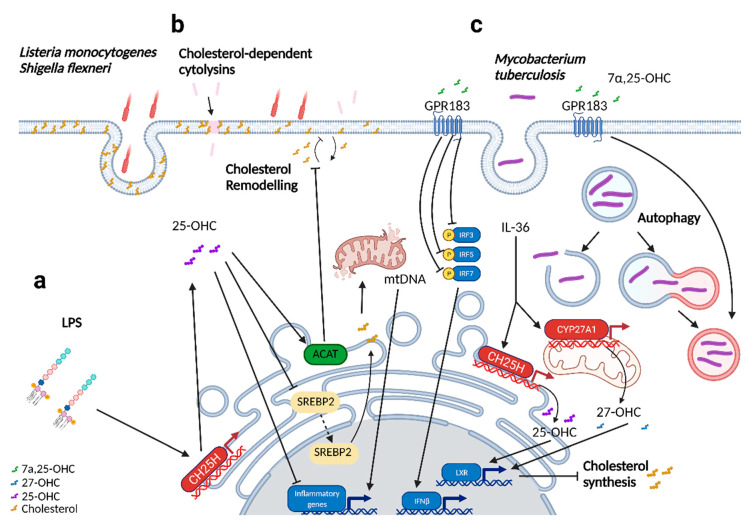
Mechanisms of oxysterol action in bacterial infections. Several oxysterols have host protective roles against bacterial pathogens. (**a**) In LPS models of infection, the anti-inflammatory role of the CH25H/25-OHC axis has been demonstrated in several macrophage models. In murine alveolar macrophages, 25-OHC administration led to a reduction in inflammation markers (TNFα, IL-6) though mechanisms yet to be elucidated. In BMDMs, 25-OHC represses cholesterol production by preventing SREBP2 translocation to the nucleus, preventing cholesterol-mediated mitochondrial dysfunction and the subsequent downstream inflammatory response. (**b**) 25-OHC prevents the bacterial entry of *Listeria monocytogenes* and *Shigella flexneri* through the ACAT-dependent remodeling of cholesterol on cell membranes. In addition, CH25H/25-OHC protects against CDCs-induced pore damage by similar mechanisms, preventing CDCs from binding to cell membranes. (**c**) In *Mycobacterium tuberculosis* (Mtb) infection, the GPR183/7α,25-OHC axis negatively regulates the type I IFN pathway and promotes autophagy, limiting mycobacterial growth in primary human monocytes. In addition, in human macrophages (THP-1s and monocyte-derived macrophages) Mtb-induced IL-36 facilitates the production of 25-OHC and 27-OHC, which inhibits cholesterol synthesis by activating LXR downstream.

In addition, the immunomodulatory function of 25-OHC against a broad range of bacterial pathogens has been proposed in studies conducted with lipopolysaccharide (LPS), a major component of the outer membrane of Gram-negative bacteria. In the lung, the anti-inflammatory role of 25-OHC against acute lung inflammation through LPS stimulation has been studied [33,34]. 25-OHC was observed to be produced upon LPS stimulation in the lungs and bronchoalveolar lavage fluid. CH25H KO mice displayed a delayed resolution of inflammation [33]. Additionally, alveolar macrophages from CH25H KO mice displayed increased cholesterol accumulation and defective efferocytosis [33]. The in vivo administration of 25-OHC led to a reduction in immune cell infiltration and inflammation [34] and accelerated the resolution of inflammation in CH25H KO mice [33]. Another study also demonstrated the anti-inflammatory role of the CH25H/25-OHC axis in vivo in LPS-stimulated BMDMs [65]. Upon LPS stimulation, 25-OHC is produced to prevent the cholesterol-dependent DNA sensor protein absent in melanoma 2 (AIM2) activation and the subsequent downstream activation of IL-1β. The CH25H/25-OHC axis prevents the translocation of SREBP2 to the nucleus for the onset of cholesterol synthesis. In addition, CH25H KO BMDMs resulted in an increased accumulation of sterols (desmosterol, lanosterol, and 7-dehydrocholesterol) involved in cholesterol biosynthesis. The increased cholesterol load in CH25H KO BMDMs was associated with impaired mitochondrial metabolism and mitochondrial dysfunction, releasing mitochondria DNA into the cytosol for the downstream activation of AIM2 and inflammatory responses [65]. A schematic overview of the known mechanisms of oxysterol action in the immune response to bacterial infections is shown in Figure 2.

## 4. Oxysterols in Viral Infections

In the context of viral infections, the immunomodulatory and antiviral activities of oxysterols have been shown by multiple studies. The majority of the literature describes the antiviral activities of 25-OHC; however, recent studies have suggested similar antiviral activities for other side-chain oxysterols such as 27-OHC and 7-Ketocholesterol (7-KC) [28]. These oxysterols have been extensively studied in many viruses, ranging from enveloped viruses such as severe acute respiratory syndrome coronavirus-2 (SARS-CoV-2) [47,66,67,68], influenza A virus (IAV) [26], human immunodeficiency virus (HIV) [27], Zika virus (ZIKV) [69], pseudorabies [70], hepatitis C virus [71], and many others [44,69,70] to non-enveloped viruses such as Seneca valley virus [29], murine norovirus [72], rhinovirus [43,73], rotavirus [46], and human papillomavirus-16 [43], among others [74,75,76].

### 4.1. Oxysterols and Viral Entry

Cellular membranes contain microdomains that are rich in cholesterols and sphingolipids called lipid rafts that are often exploited by viruses, both enveloped and non-enveloped viruses, for their viral life cycle [77]. Many viruses utilize these cholesterol-rich regions for internalization [78] and entry into the host cell through a variety of mechanisms ranging from curvature formation to receptor clustering and binding to viral fusion proteins to gain entry. This, hence, highlights the need for cholesterol for efficient entry into cells, although it is noted that not all viruses depend on lipid rafts for entry [77]. In this regard, the CH25H/25-OHC axis has shown to inhibit viral entry through modifying the cholesterol composition on the plasma membrane, preventing membrane fusion for enveloped viruses [27,46,49,66,67,68,69,70,73,79] and altering endosomal dynamics and its cholesterol composition, thereby preventing cytosolic entry for non-enveloped viruses [46,74]. The majority of the research conducted so far has demonstrated the potent antiviral activity of 25-OHC in inhibiting viral entry. Although other oxysterols such as 22(S)-OHC, 20α-OHC, and 7β-hydroxycholesterol (7β-OHC) can also inhibit viral entry, their molecular mechanisms of action have not been fully characterized [49].

Several mechanisms of action for 25-OHC regarding inhibiting the viral entry of many enveloped viruses—for instance, Porcine reproductive and respiratory syndrome virus [79]; hepatitis B virus [49]; ZIKV [69]; and, more recently, SARS-CoV-2 [30,68]—have been suggested. The majority of these studies demonstrate that the alteration of cholesterol on the plasma membrane is the key to restriction for viral entry, although various studies have demonstrated that 25-OHC is able to be localized within the plasma membrane [27], directly affecting membrane properties [31,80]. Mechanistically, infection studies with SARS-CoV-2 have elucidated that 25-OHC induces the depletion of cholesterol on the plasma membrane through ACAT activation [68]. This observation is consistent with work on intracellular pathogens, whereby cholesterol remodeling on the plasma membrane upon infection is caused by the activation of ACAT [31]. Therefore, this mode of action appears to be conserved across viral and bacterial infections [31,68]. In addition, the benefits of utilizing 25-OHC in combination with other viral inhibitors have been studied recently on human coronaviruses [30]. The authors conjugated 25-OHC with a peptide-based viral inhibitor with a different mode of action and tested its inhibitory efficacy against a broad range of coronaviruses. The resulting 25-OHC-conjugated lipopeptide (EK1P4HC) demonstrated a synergistic antiviral effect on inhibiting viral entry against SARS-CoV-2 and its variants as well as other human coronaviruses [30]. Apart from inducing cholesterol remodeling in plasma membranes, 25-OHC also localizes within late endosomes, where it prevents SARS-CoV-2-mediated entry to the cytosol through inhibiting cholesterol export [67].

The antiviral effects of 25-OHC have also been studied in vivo, with studies demonstrating that the administration of 25-OHC is protective against HIV [27], ZIKV [69], and SARS-CoV-2 [66], and transgenic KO studies showing that CH25H deficiency results in an increased susceptibility to murine gammaherpesvirus 68 (MHV68) [27].

Apart from 25-OHC, 27-OHC has also been gaining attention for having broad antiviral activity against several viruses, such as murine cytomegalovirus [26], human papillomavirus [43], rhinoviruses [43], herpes simplex-1 virus [45], rotavirus [46], and SARS-CoV-2 [47], among others [43]. Mechanistically, in vitro studies suggest that the antiviral activity of 27-OHC is multifactorial, including interfering with viral entry into the cell, inducing cholesterol remodeling on plasma membranes and endosomes, and regulating adhesion molecules [42] and pro-inflammatory cytokine production [45].

A recent study highlighted the importance of 27-OHC in SARS-CoV-2 infection. Consistent with previous experiments conducted on other viruses, the in vitro administration of 27-OHC prior to infection reduced the intracellular accumulation of SARS-CoV-2 as well as human coronavirus OC-43. Furthermore, using mass spectrometry analysis, the authors demonstrated that the serum 27-OHC levels of patients were inversely correlated with the disease severity of COVID-19 [47].

Among non-enveloped viruses, a study conducted on human rotavirus demonstrated that 25-OHC and 27-OHC alter the endosomal dynamics in MA104 cells through preventing the interaction between oxysterol binding protein (OSBP) and vesicle-associated membrane protein-associated protein-A (VAP-A) [46]. This study demonstrated that these oxysterols are able to displace OSBP from the ER to the Golgi, preventing its interaction with VAP-A. The OSBP-VAP-A complex regulates cholesterol transport from the ER to intracellular organelles such as the endosomes. The disruption of the complex by these oxysterols thereby prevents cholesterol recycling between the ER and the late endosomes. This, in turn, results in the accumulation of cholesterol within the late endosomes and inhibits rotavirus entry into the cytoplasm [46]. Similarly, in reovirus infections 25-OHC alters endosomal dynamics upon infection. However, the authors suggested an alternative mechanism of restriction induced by 25-OHC on reovirus-infected cells. They demonstrated that, in HeLa cells, 25-OHC reduces the co-localization of the viral particles with the late endosomal marker Rab7. As reovirus entry into the cell is dependent on the timely uncoating of the virus in the late endosomes, the authors suggested that the main mechanism of restriction is due to this delayed trafficking of the viral particles to the late endosomes, which alters the uncoating of the virus and its subsequent penetration efficiency into the cell cytoplasm [74]. In Seneca valley virus infection, a study highlighted that CH25H activity is inversely correlated with viral replication and that 25-OHC inhibits Seneca valley virus replication in a dose-dependent manner in HEK-293T and BHK-21 cells [29]. The authors further elucidated that 25-OHC specifically inhibited the viral absorption process of the viral life cycle, with no effect observed on the later stages of the viral replication cycle [29].

### 4.2. Oxysterols and Restriction of Viral Replication

In addition to the preventing viral entry, the antiviral effects of 25-OHC extend further to modifying cholesterol content intracellularly and restricting viral replication [75]. In vitro, both the pre- and post-treatment of Poliovirus pseudovirus-infected HEK293 cells with 25-OHC was found to reduce viral replication. The authors further demonstrated that 25-OHC interacts with OSBP, leading to the reduced accumulation of Phosphatidylinositol 4-phosphate (PI4P) at the golgi apparatus [75,81]. PI4P has been implicated in supporting poliovirus replication partially through the recruitment of unesterified cholesterol to PV-induced membrane structures [81]. Hence, the reduction in PI4P induced through the 25-OHC/OSBP axis might reduce the cholesterol availability on PV-induced membranes that is required for replication.

Another mechanism of the antiviral activity of 25-OHC has been suggested to be the upregulation of the integrated stress response pathway. Authors found that the endogenous production of 25-OHC in BMDMs during MCMV infection leads to the induction of stress response genes independently of LXRs [82]. Furthermore, the addition of 25-OHC in BMDMs leads to the activation of general control nonderepressible 2 (GCN2), one of the eIF2α kinases that senses and activates the integrated stress response pathway [82]. Thus, the activation of the integrated stress response could lead to the suppression of protein synthesis, which viruses depend on for viral replication [82].

A cell-based screening of oxysterols in SARS-CoV-2-infected TMPRSS2-overexpressed VeroE6 cells identified 7-KC, 22R-hydroxycholesterol (22(R)-OHC), 24S-hydroxycholesterol (24S-OHC), and 27-OHC as potent inhibitors of SARS-CoV-2 replication in vitro [44]. In addition to these natural oxysterols, the authors further demonstrated that the semi-synthetic oxysterol derivatives Oxy210 and Oxy232 have a higher antiviral potency than the natural oxysterols. They further elucidated that Oxy210 inhibits the in vitro replication of SARS-CoV-2 and HCV through reducing double membrane vesicles (DMVs)-dependent replication [44]. These existing findings place oxysterols and their synthetic analogues in the spotlight as novel therapeutics for infectious diseases.

### 4.3. Oxysterols in Viral Assembly and Release

Another antiviral role of 25-OHC has been suggested in Lassa virus (LASV) infections, whereby 25-OHC was able to interfere with the late stages of the LASV life cycle through interfering with the viral glycosylation, affecting the production of infectious viruses [83]. In vitro, 25-OHC alters the glycosylation of the LASV glycoprotein 1 (GP1) in Huh-7 cells, causing an increased presence of immature forms of *N*-glycans on GP1 and thereby leading to the production of less infectious virus progeny which have defective entry. Furthermore, the overexpression of CH25H affected GP1 glycosylation, and the infectious viral production and knockdown of CH25H led to an increased production of infectious LASV, further supporting the importance of the CH25H/25-OHC axis in LASV infection [83].

**Figure 3:**
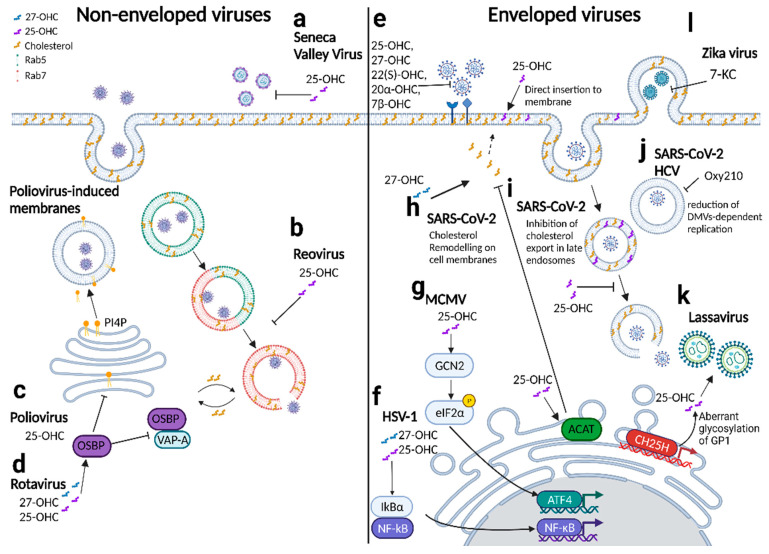
Mechanisms of oxysterol action in viral infections. Oxysterols have broad antiviral activities across both non-enveloped viruses (left) and enveloped viruses (right). Among non-enveloped viruses, (**a**) 25-OHC inhibits the absorption of Seneca valley virus in BHK-21 cells without affecting the other stages of the viral replication cycle. (**b**) 25-OHC regulates endosomal dynamics in reovirus infection by reducing the co-localization of viral particles with Rab7 in HeLa cells. (**c**) In poliovirus pseudovirus-infected HEK293 cells, 25-OHC reduces viral replication by interacting with OSBP to reduce PI4P accumulation on poliovirus-induced membranes. (**d**) In rotavirus-infected MA104 cells, 25-OHC and 27-OHC interact with OSBP to prevent its subsequent interaction with VAP-A. This results in reduced cholesterol recycling between the ER and late endosomes, thereby sequestering viral particles within late endosomes and preventing cytosolic entry and replication. Among enveloped viruses, (**e**) the oxysterols 25-OHC, 27-OHC, 22(S)-OHC, 20α-OHC, and 7β-OHC prevent viral entry across a broad range of viruses, some through mechanisms have not yet been elucidated. 25-OHC has been shown to co-localize within the plasma membrane, affecting membrane properties and preventing viral entry. (**f**) In HSV-1-infected, cells, 25-OHC and 27-OHC regulate the inflammatory response by inducing NF-κB activation, promoting the upregulation of inflammatory genes involved in antiviral control. (**g**) In MCMV-infected BMDMs, 25-OHC activates the integrated stress response pathway for its antiviral functions. (**h**) In SARS-CoV-2-infected HEK293-hACE2 and VeroE6 cells, 27-OHC and 25-OHC induce cholesterol remodeling on the plasma membrane, preventing viral entry. (**i**) Additionally, 25-OHC is also able to localize within late endosomes, where it inhibits cholesterol export, preventing SARS-CoV-2-mediated membrane fusion for cytosolic entry. (**j**) In SARS-CoV-2 and HCV infections, oxy210 displayed antiviral activities by limiting the viral-induced DMV-dependent replication. (**k**) 25-OHC affects glycoprotein glycosylation and the production of infectious virions in LASV-infected huh-7 cells. (**l**) In ZIKV-infected cells, 7-KC prevents viral budding from host cells, decreasing the viral progeny production.

Through an autophagy compound screening in ZIKV-infected Vero and C6/36 cells, one group identified an antiviral role of 7-KC in ZIKV infections [55]. The in vitro administration of 7-KC interfered with the later stages of the viral life cycle, as characterized by a reduction in the viral budding efficiency and infectious virion production, with no impact on viral entry or intracellular viral replication. Although the exact mechanism has yet to be elucidated, the authors suggest that 7-KC could influence the intracellular lipid environment in the organelles involved in ZIKV trafficking [55]. A summary overview of the known mechanisms of oxysterol action in the immune response to viral infections is shown in Figure 3.

## 5. Oxysterols in Disease Pathogenesis and as Potential Biomarkers

Apart from their antiviral activities, several studies have also implicated oxysterols such as 7-KC as being detrimental in severe viral infections. While 7-KC has been demonstrated to have antiviral activities across several different viruses in vitro, in patients, however, a pathological role of 7-KC has been suggested with several viral pathogens due to its known cytotoxic properties at high concentrations [56]. 7-KC promotes a pro-inflammatory phenotype in human macrophages [53] and may thus, together with other oxysterols, contribute to excessive inflammation. Several studies have demonstrated the cytotoxic effects of these oxysterols among several non-immune cells, such as endothelial cells [84], neuronal cells [85], and mesenchymal stem cells [86,87]. Mechanistically, a study conducted on human umbilical vein endothelial cells (HUVECs) found that 7-KC and 7β-OH drive endothelial dysfunction by inducing early lipid accumulation and lysosomal permeabilization [84]. This results in increased oxidative stress, leading to apoptosis in these cells [84]. Other mechanisms, such as mitochondrial hyperpolarization [85] and changes in actin polarization, caspase activation, and autophagy, have also been described [86,87]. In viral infections, the plasma concentrations of 7-KC were elevated following human herpesvirus 8 infection [57] in diabetes patients and patients with influenza [51], as well as patients with COVID-19 [50]. In COVID-19, an increase in 7-KC is observed in patients with moderate and severe COVID-19, but this rises gradually along with disease severity. It has been proposed that the pro-inflammatory and cytotoxic effects of 7-KC contribute to the cytokine storm and acute respiratory distress and that 7-KC is thus involved in disease progression and poor outcomes [50]. Consequently, 7-KC may therefore serve as a biomarker for COVID-19 severity [50]. 7-KC has also been implicated in driving disease pathogenesis in cardiovascular diseases [56], and oxysterols have also been proposed as potential biomarkers for chronic and neurodegenerative diseases [88]. The translational potential of oxysterol research and the therapeutic application of oxysterols for viral infections and other chronic diseases are gaining momentum [88].

## 6. Conclusions and Future Perspectives

In this review, we highlighted an emerging field of research: oxysterols as important immunomodulators of infectious diseases. Initially thought to only be involved in cholesterol homeostasis, many studies conducted throughout the past decade have described the important role of oxysterols in both physiological and pathological conditions. Oxysterols are produced as part of the host immune response towards several bacterial and viral infections. In addition, the mechanisms of action induced by these immune oxysterols are broad, ranging from chemotaxis and regulating inflammatory responses to restricting intracellular cholesterol content, some of which are conserved across viral and bacterial pathogens. Given the increasing prevalence of antimicrobial resistance and the emergence of novel viruses, there is an increasing need to find new and innovative strategies to combat these pathogens. The growing evidence of the role of oxysterols in contributing to the immune response to infections hence presents them as novel biomarkers of disease severity and suggests oxysterol receptors to be attractive targets for host-directed therapy for improving bacterial and viral infectious disease outcomes.

## Figures and Tables

**Table 1:** Selected oxysterols and their receptors. Oxysterols are known to bind to the receptors listed in this table; however, in some cases it remains to be confirmed through which receptor the immune modulatory effects are mediated.

Oxysterol	Synthesizing Enzyme	Molecular Targets/Receptor	Immunomodulatory Effects	References
25-hydroxycholesterol (25-OHC)	Synthesized from cholesterol by CH25H; Autoxidation from cholesterol	LXRs [21] RORα [22] RORγt [23] INSIGs [11] GPR183 [18,24] ERα [25]	Produced by macrophages upon viral infection to mediate antiviral functions; broad antiviral activity against enveloped and non-enveloped viruses.	[26,27,28,29,30]
			Triggers cholesterol remodeling on the plasma membrane, restricting the intracellular dissemination of *Listeria monocytogenes* and *Shigella flexneri*; prevents CDC-induced pore damage.	[31,32]
			Produced upon lipopolysaccharide (LPS) stimulation in the lungs. CH25H was found to be upregulated up to 24 h post-infection. Pulmonary administration of 25-OHC resulted in reduced immune cell infiltration and inflammation in the lung.	[33,34]
			Downregulated upon exposure to house dust mites. CH25H was found to be upregulated in contrast. Pulmonary administration of 25-OHC resulted in a more severe onset of inflammation and airway remodeling.	[34]
7 α25-dihydroxycholesterol (7α,25-OHC)	Converted from 25-OHC by CYP7B1	GPR183 [18,24] RORγ [19]	In vitro modulation of mycobacterial growth in primary macrophages induces autophagy and regulated inflammatory responses, including type 1 interferons. In RAW264.7 cells, CH25H and CYP7b1 expression was downregulated at 24 h post-infection.	[35,36]
			Mediates the proper positioning of immune cells (ILC3s, Dendritic cells, T_FH_, B cells) to their respective niches.	[15,16,17,18,37]
27-hydroxycholesterol (27-OHC)	Synthesized from cholesterol by CYP27A1	INSIGs [11] LXRs [38] ERα [39] ERβ [40] RORγ [19] GPR17 [41]	Antiviral activity against enveloped and non-enveloped viruses; reduced in the serum of SARS-CoV-2 patients.	[26,42,43,44,45,46,47]
7α,27-dihydroxycholesterol (7α,27-OHC)	Converted from 27-OHC with the help of CYP7B1	RORγt [16] GPR183 [18,24]	Induces IL-17 production in Th17 cells, aids in Th17 cell differentiation.	[19]
7β-hydroxycholesterol (7β-OHC)	Autoxidation from cholesterol	RORα [48] RORy [48]	Antiviral activity against hepatitis B virus; Elevated in the serum of COVID-19 patients; Elevated in plasma of Influenza patients.	[47,49,50,51]
7-Ketocholesterol (7-KC)		LXRs [21] RORα [48] RORy [48] ERα [52]	Pro-inflammatory and cytotoxicity effect of 7-KC could possibility lead to cytokine storms; promotes a pro-inflammatory macrophage phenotype. Affects the polarization of macrophages.	[50,53,54]
			Antiviral activity in vitro against SARS-CoV-2 and ZIKV.	[44,55]
			Implicated in chronic diseases (atherosclerosis, Alzheimer’s disease).	[56]
			Elevated in the serum of COVID-19 patients; Elevated in the plasma of human herpesvirus-8 and Influenza patients.	[47,50,51,57]
24S-hydroxycholesterol (24S-OHC)	Synthesized from cholesterol by CYP46A1	INSIGs [11]	Antiviral effects against murine cytomegalovirus.	[26]
			Antiviral activity in vitro against SARS-CoV-2 replication.	[44]
22R-hydroxycholesterol (22R-OHC)	Synthesized from cholesterol by CYP11A1	INSIGs [11] LXRs [21] RORγ [23] CXCR2 [58] GPR17 [41]	Antiviral activity in vitro against SARS-CoV-2 replication.	[44]
